# Enhanced Self-Organized Dewetting of Ultrathin Polymer Blend Film for Large-Area Fabrication of SERS Substrate

**DOI:** 10.1038/srep38337

**Published:** 2016-12-06

**Authors:** Huanhuan Zhang, Lin Xu, Yabo Xu, Gang Huang, Xueyu Zhao, Yuqing Lai, Tongfei Shi

**Affiliations:** 1 State Key Laboratory of Polymer Physics and Chemistry, Changchun Institute of Applied Chemistry, Chinese Academy of Sciences, Changchun 130022, P. R. China; 2 Laboratory of Surface Physics and Chemistry, Guizhou Education University, Guiyang 550018, P. R. China; 3 University of Chinese Academy of Sciences, Beijing 100049, P. R. China; 4 School of Chemistry and Life Sciences, Guizhou Education University, Guiyang 550018, P. R. China

## Abstract

We study the enhanced dewetting of ultrathin Polystyrene (PS)/Poly (methyl methacrylate) (PMMA) blend films in a mixed solution, and reveal the dewetting can act as a simple and effective method to fabricate large-area surface-enhanced Raman scattering (SERS) substrate. A bilayer structure consisting of under PMMA layer and upper PS layer forms due to vertical phase separation of immiscible PS/PMMA during the spin-coating process. The thicker layer of the bilayer structure dominates the dewetting structures of PS/PMMA blend films. The diameter and diameter distribution of droplets, and the average separation spacing between the droplets can be precisely controlled via the change of blend ratio and film thickness. The dewetting structure of 8 nm PS/PMMA (1:1 wt%) blend film is proved to successfully fabricate large-area (3.5 cm × 3.5 cm) universal SERS substrate via deposited a silver layer on the dewetting structure. The SERS substrate shows good SERS-signal reproducibility (*RSD* < 7.2%) and high enhancement factor (2.5 × 10^7^). The enhanced dewetting of polymer blend films broadens the application of dewetting of polymer films, especially in the nanotechnology, and may open a new approach for the fabrication of large-area SERS substrate to promote the application of SERS substrate in the rapid sensitive detection of trace molecules.

Surface-enhanced Raman scattering (SERS) has drawn attention for broad applications and scientific interests in the fields of analytical chemistry^1^, sensing^2^,^3^, molecular biology^4^, food science^5^ and environmental science^6^,^7^ so on. For SERS-based application, intensive efforts have been devoted to fabricate the high effective SERS substrates consisting of noble metal (Au or Ag) materials and building blocks during the last two decades. The electromagnetic field around noble metallic nanostructures can be enhanced via localized surface plasm on resonance (LSPR) of noble metallic nanostructures^8^,^9^. The size of nanostructures and interparticle gap spacing strongly influence the property of SERS substrates^10^,^11^. Although some flexible nanofabrication approaches like nanoimprinting lithograph^12^,^13^, nanosphere lithograph^14^,^15^ and electron or ion-beam lithography^16^,^17^, have had great success in producing the SERS substrate with excellent properties, these methods are expensive, time-cost or not suitable for large-area application. These shortcomings limit the application of these methods. Thus, it is still a great challenge to fabricate large-area effective SERS substrates via simple and low-cost methods.

Alternatively, the simple dewetting of thin polymer films has the promising potential for application in the fabrication of the large-area SERS substrate. As a simple and low-cost bottom-up approach, self-organized dewetting of thin polymer films has attracted considerable attention in recent years^18^,^19^,^20^,^21^,^22^,^23^,^24^,^25^,^26^,^27^,^28^. Dewetting on a defect free homogeneous substrate progresses with the formation and growth of randomly placed holes with a characteristic length scale. The earlier works reveal that the average size of the dewetted structures as well as their dominant wavelength depend on the initial film thickness and surface tension of polymer film^29^,^30^,^31^,^32^,^33^,^34^,^35^,^36^. The size of dewetted structure determines the application of dewetting in different fields. In generally, the thermal-induced dewetting and solvent vapor-induced dewetting of polymer films are mainly used to produce micrometer patterning due to the relative high surface tension between polymer and air or solvent vapor^37^,^38^,^39^. Miniaturization of the instability length scale to smaller size remains a major challenge. Recently, Sharma *et al*. proposed an attractive technique for the control of instability by annealing thin polymer film under a homogeneous liquid mixture, which can engineer polymeric domains of nanoscale or micron-size^40^. The unusual dewetting method successfully reduces the instability length scale to nanometer size. However, another limitation for application in nanotechnology of the simple dewetting method is that it produces droplets with relative big diameter, wide diameter distribution and relative larger mean separation spacing between the droplets.

Here, we propose a very simple approach (the dewetting of thin polymer blend film in a mixed solution) to solve these limitations for dewetting of single polymer film. We change the ratio of immiscible polymers (PS/PMMA) in the films to precisely control the diameter of droplets, diameter distribution of droplets and the average separation spacing between the droplets. It is worth mentioning that the enhanced dewetting of thin polymer blend films broadens the application of dewetting of polymer films, especially in the nanotechnology. The dewetting structure of thin polymer blend films in the mixed solution is demonstrated to be successful in being the building block of SERS substrate. Once a thin silver film is deposited on the dewetting structures of 8 nm PS/PMMA (1:1 wt%) blend films, we can fabricate large-area (3.5 cm × 3.5 cm), high efficient (enhancement factor *EF* > 2.5 × 10^7^) and good SERS-signal reproducibility (*RSD* < 7.2%) SERS substrate, which is very convenience in fabrication and manipulation.

## Results and Discussion

**Figure 1:**
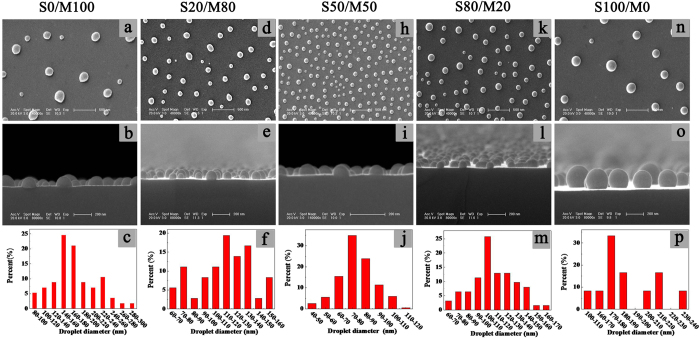
SEM images and diameter distribution of final dewetting droplets of PS/PMMA blend films (h = 8 nm). The top view images (the first row), cross-section view (the second row) images and diameter distribution (the third row) of final droplets are shown. The films were put in the mixed solution of methyl ethyl ketone (MEK), acetone and water in the ratio 7:3:35 for 30 minutes to induce dewetting at room temperature. (**a**–**c**) S0/M100 film; (**d**–**f**) S20/M80 film; (**h**–**j**) S50/M50 film; (**k**–**m**) S80/M20 film film; (**o**,**p**) S100/M0 film. The scale bar in top view images and cross-section view images is 500 nm and 200 nm, respectively.

A series of ultrathin PS/PMMA blend films are immersed in mixed solution. The diffusion of solvent reduces the glass transition temperature of polymer blend films below to room temperature^32^,^40^. At the action of excess intermolecular forces or electrostatic forces, the dewetting of blend films is engendered. The mixed solution cannot dissolve the PS and PMMA because the morphology of droplets keeps the same over 24 h in the mixed solution. Figure 1 shows the final dewetting morphology of 8 nm PS/PMMA blend films in mixed solution and the diameter distribution of droplets. As shown in Fig. 1, the diameter of droplets firstly decreases, and then increases with the increases of PS weight fraction (wt%). When the PS/PMMA blend ratio is 1:1 (referred to S50/M50 film, for simplicity), the mean diameter of droplets is smallest and the diameter distribution of droplets is narrowest. Supplementary Fig. S1 shows the final dewetting morphology of PS/PMMA blend films with different thicknesses. From Supplementary Fig. S1, it is found that the diameter of droplets can be tuned from sub-μm to sub-50 nm and the mean separation spacing of the droplets also can be adjusted from sub-μm to sub-100 nm via the dewetting of thin polymer blend films in mixed solution.

**Figure 2:**
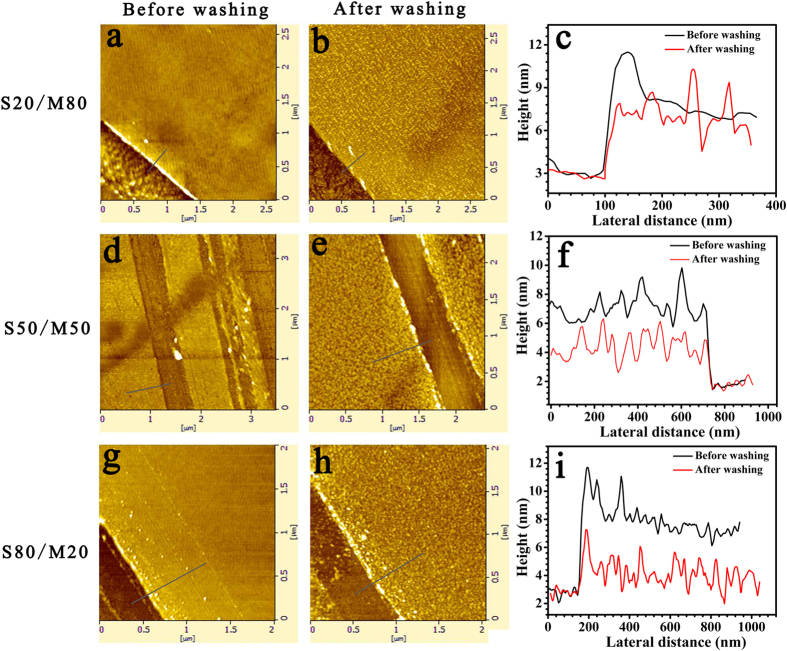
AFM images of different polymer blend films of 8 nm. The first column are the AFM images of original polymer blend film before washing by cyclohexane. To remove PS in the blend films, the blend films were washed by cyclohexane. The second column show the AFM images of polymer blend film after washing by cyclohexane. The third column are sectional views along the line (the gray line) in first and second column. (**a**–**c**), (**d–f**) and (**g**–**i**) the films are S20/M80, S50/M50 and S80/M20, respectively.

In order to further understand the dewetting of thin PS/PMMA blend films in the liquid mixture, the structure of PS/PMMA blend films before dewetting and the structure of droplets are investigated firstly. Figure 2 presents us the morphologies of 8 nm PS/PMMA blend films obtained by AFM. Figure 2a–c imply that the S20/M80 film becomes much coarser and the film thickness decreases slightly after washing by cyclohexane which is selective solvent for PS. As indicated in Fig. 2d–f, the thickness of S50/M50 decreases by half after washing by cyclohexane. The thickness of S80/M20 decreases much more obviously and the roughness increases obviously after washing by cyclohexane (see Fig. 2g–i). As we scratch the PS/PMMA blend films by tweezers and then wash the same films using acetic acid which is selective solvent for PMMA, the scratch disappears and no film is still residual on the silicon wafer. These results indicate the PS/PMMA blend films undergo vertical phase separation and form a bilayer structure (the under layer is PMMA and the upper layer is PS) during the spin-coating process. In order to further prove the formation of bilayer structures during spin-coating process, the PS/PMMA/Si substrate bilayer film is fabricated (see Supplementary Fig. S2a).

**Figure 3:**
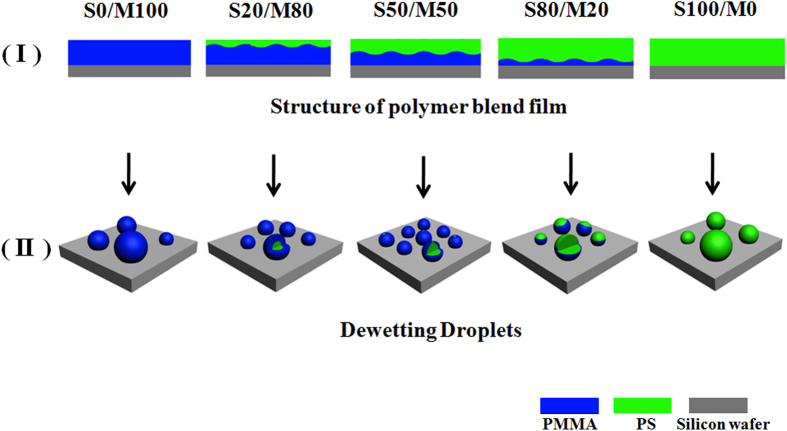
Schematic diagrams of polymer blend films and droplets. (Ι) The cross-section schematic diagrams of polymer blend films. The PS/PMMA undergoes vertical phase separation and form a bilayer structure during the spin-coating process. The under layer is the PMMA-rich layer, the upper layer is the PS-rich layer. With the increase of the PS weight fraction in polymer solution, the thickness of the PS-rich upper layer increases and the thickness of the PMMA-rich under layer decreases. (ΙΙ) The schematic diagrams of the final dewetting droplets. The structure of droplet is core-shell structure consisting of the PS core and the PMMA shell. As PS fraction is little, such as 20%, the PMMA shell can wrap the PS core completely. As PS fraction is much, for example 80%, the PMMA shell cannot wrap the PS core completely. The less the PS fraction in the film is, the more obvious the core-shell structure is.

As shown in Fig. 2d–f and Supplementary Fig. S2b–d, the structure of S50/M50 film is similar to the structure of bilayer film. The dewetting results of bilayer film are shown in Supplementary Fig. S3. Comparing the S50/M50 blend film with the bilayer film, their dewetting processes and final morphologies are almost same as their thicknesses are same. The dewetting results of bilayer film further support the above results of AFM image. The schematic diagram for bilayer structure of polymer blend films before dewetting are shown in Fig. 3I.

From thermodynamics aspect, the interaction between polymer and solvent is indicated by the Flory-Huggins interaction parameter (also known as *χ* parameter). The Flory-Huggins interaction parameter of polymer-solvent system *χ*_*p-s*_ is expressed by^41^,^42^:


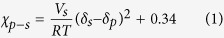


where *V*_*s*_ is the molar volume of the solvent, *R* is the universal gas constant, *T* is the temperature, and *δ*_*p*_ and *δ*_*s*_ are the solubility parameters of the polymer and solvent respectively. The smaller *χ*_*p-s*_ indicates the higher interaction between the polymer and the solvent^41^. The *δ* values of PS, PMMA and toluene are 18.6, 19.2 and 18.2 MPa^1/2^, respectively^42^,^43^. Based on equation (1), the *χ*_*p-s*_ of PS and toluene is smaller than *χ*_*p-s*_ of PMMA and toluene, which implies that toluene is better solvent for PS than for PMMA. Besides, the PMMA are preferentially adsorbed on the silicon wafer due to a fairly strong attractive interaction between carbonyl groups of PMMA and the silanol groups on the silicon wafer^44^. According to the above thermodynamic analysis, the PMMA solidifies firstly from toluene solution of polymer and forms the PMMA-rich under layer on the silicon wafer during the spin-coating process^45^,^46^. After the PMMA under layer formed, the solution keeps evaporating and solidifying to form the PS-rich upper layer^47^,^48^. With the increase of the PS weight fraction in polymer solution, the thickness of PS-rich upper layer increases and the thickness of PMMA-rich under layer decreases. The above results of bilayer structure can be explained via the thermodynamic analysis.

In order to have an insight into the structures of droplets, the selective solvents are used to etch the droplets. Firstly, we use the PMMA selective solvent (acetic acid) to etch the droplets. As shown in Supplementary Fig. S4, in the case of 8 nm S80/M20 film, the average diameter of droplets decreases slightly and some droplets can aggregate together (Supplementary Fig. S4b). In the case of 8 nm S50/M50 film, the diameter of droplets becomes smaller and the morphologies of rod and star are found (Supplementary Fig. S4e); in the case of 8 nm S20/M80 film, the diameter of droplets obviously decreases, the droplets aggregate again and the number of droplets also significantly reduces (Supplementary Fig. S4h). According to the results of etching process, we can deduce that PMMA enriches at the surface of droplets. Because the acetic acid is good solvent of PMMA, PMMA is removed from the droplet during the etching process. And the decrease of droplet diameter confirms that the outside of the droplet is PMMA. After PMMA shell is removed, the move of the residual PS droplet is easier. In addition, in order to reduce the energy of system, the residual PS droplets trend to reduce the droplet surface. Thus, the residual droplet tends to aggregate together again when they are etched by acetic acid. During the etching process of the PS selective solvent-cyclohexane, it is found that in the case of 8 nm S80/M20 film, many droplets are etched into irregular shape and the diameter of many droplets decreases obviously (see Supplementary Fig. S4c); in the case of 8 nm S50/M50 film, some droplets become flat and small holes appear on some droplets (Supplementary Fig. S4f); and in the case of 8 nm S20/M80 film, the morphologies of droplets hardly change (Supplementary Fig. S4i). Based on the results of etching process, we can deduce that the structure of droplet is a PS core and PMMA shell structure. Supplementary Fig. S5a–c show the result of etching result of 27 nm PS/PMMA blend film. These results more clearly reveal the core-shell structure. The schematic diagram of the structures of droplets in different PS/PMMA blend films is shown in Fig. 3II. The droplets consist of the PS core and the PMMA shell. As PS fraction is little, for instance 20%, the PMMA shell can wrap the PS core completely. As PS fraction is much, for example 80%, the PMMA shell cannot wrap the PS core completely. In order to study the droplet formation mechanism, the structure of the ribbon is investigated. As the ribbon is etched by cyclohexane (the selective solvent of PS), we find that PS distributes in the middle of the ribbon and PMMA distributes on the three phase contact line (see Supplementary Fig. S6). In addition, from Supplementary Fig. S4, it is found that the structure of droplet is a PS core and PMMA shell structure. According to these results, when the blend film is treated in mixed solvent, firstly the upper PS-rich layer ruptures because interfacial tension PMMA-PS is low, which allows easy destabilization of the interface; and then under PMMA-rich layer breakups; finally PMMA wraps PS to form the structure of core-shell.

**Figure 4:**
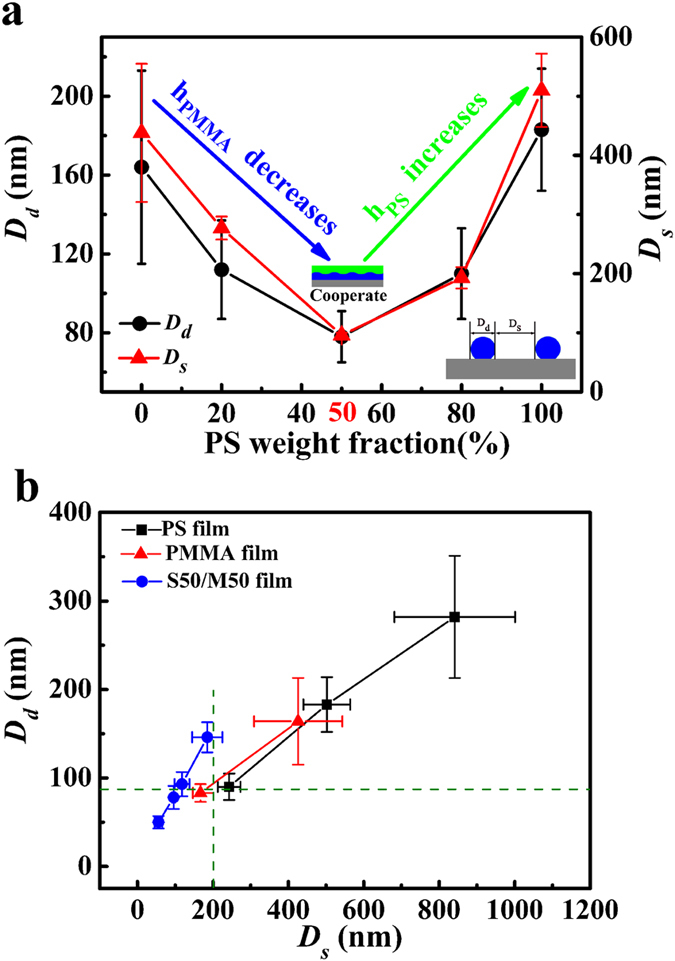
The average droplet diameter *D*_*d*_ and mean droplet separation spacing *D*_*s*_. (**a**) The dependence of *D*_*d*_ and *D*_*s*_ on blend ratio. The original thicknesses of all films are 8 nm. *h*_*PMMA*_ is the thickness of PMMA layer and *h*_*PS*_ is the thickness of PS layer, (**b**) The dependence of *D*_*d*_ on *D*_*s*_ for single polymer film and polymer blend film. The green dash lines are guides to eyes.

Based on the FESEM images, we can get the mean diameter of droplets (*D*_*d*_), and mean separation spacing between the droplets (*D*_*s*_). Figure 4a shows the change of *D*_*d*_ and *D*_*s*_ with the blend ratio. From Fig. 4a, nonmonotonic changes in *D*_*d*_ and *D*_*s*_ with increasing the PS weight fraction are witnessed. As the PS weight fraction is 50% (or blend ratio is 1:1), *D*_*d*_ and *D*_*s*_ are smallest (see Fig. 4a). In the case of bilayer system, the Gibbs free energy (Δ*G*) for the upper layer and the under layer are given by^49^,^50^,^51^,^52^:

For the upper layer:


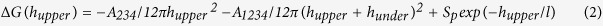


For the under layer:


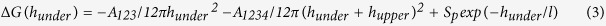


where *h*_*upper*_ is the film thickness of the upper layer, and *h*_*under*_ is the film thickness of the under layer, and *A*_*123*_, *A*_*234*_, and *A*_*1234*_ are the effective Hamaker constant (substrate-1, under layer-2, upper layer-3, and bounding medium-4); *S*_*P*_ is the polar component of spreading coefficient, and *l* is the correlation length, which is often taken as 2.78 nm.

The characteristic length scale *λ*, i.e. the mean separation spacing between the droplets, in thin film is governed by a competition between the destabilizing interface interactions and stabilizing interfacial tensions^32^:


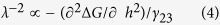


We have earlier shown that owing to closely matched van der Waals properties of PS and organic solutions, the net apolar van der Waals force in such systems is negligible or even slightly stabilizing^53^. The genesis of thin polymer film instability is thus traced to the polar interactions induced by the presence of organic solvent. Although quantitative evaluations are not possible because there is no direct method to quantify the apolar and polar interactions in this complex multicomponent system with likely interfacial segregation of some components, the qualitative picture is fairly clear. According to equation (2), the initial thickness of the polymer film is a main parameter to determine the mean separation spacing between the droplets for whatever the dewetting mechanism is. During spin-coating process, the PS/PMMA films form the bilayer structure. Thickness of each layer in the blend film is smaller than thickness of the corresponding pure PS or PMMA films. In the case of S20/M80 film, the PMMA-rich layer is thicker and it dominates *D*_*d*_ and *D*_*s*_. In the case of S80/M20 film, the PS-rich layer is thicker and it dominates *D*_*d*_ and *D*_*s*_. However, in the case of S50/M50 film, both thicknesses of the PS-rich layer and the PMMA-rich layer are close to half of the blend film thickness so that the thickness dominating *D*_*d*_ and *D*_*s*_reaches minimum. Based on the above analysis, *D*_*d*_ and *D*_*s*_ are smallest in the case of S50/M50 film. The dewetting of polymer blend film in mixed solution can be used as a simple and efficient method to control *D*_*d*_ and *D*_*s*_. Compared with the dewetting of single polymer film in mixed solution, the dewetting of polymer blend film can obtain the nanostructures with narrower diameter distribution, and smaller *D*_*s*_ at the almost same *D*_*d*_, or the bigger *D*_*d*_ at the same *D*_*s*_. (see Figs 1 and 4b).

**Figure 5:**
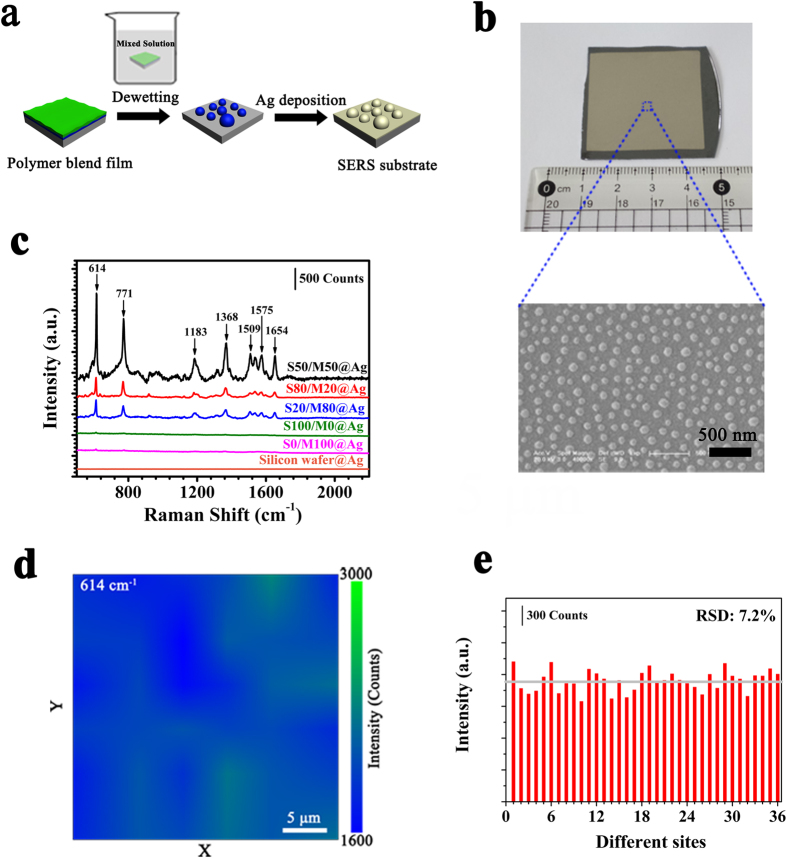
The fabrication and properties of SERS substrate. (**a**) Schematic diagram of the fabrication process of SERS substrate; (**b**) optical images of 3.5 cm × 3.5 cm large area 8 nm S50/M50@Ag SERS substrate, the SEM image is the local morphology of this SERS substrate; (**c**) SERS spectra of R6G (10^−6 ^M) on the different SERS substrate. The original thicknesses of polymer films are 8 nm; (**d**) the two-dimensional intensity mapping result at 614 cm^−1^ of R6G (10^−6 ^M) on 8 nm S50/M50@Ag substrate; (**e**) Raman intensity at 614 cm^−1^ of R6G (10^−6 ^M) acquired from 36 different spots in (**d**).

In the work, we can precisely adjust the diameter of droplets, diameter distribution of droplets and the average separation spacing between the droplets via the simple dewetting method of ultrathin PS/PMMA blend films. The SERS substrate is fabricated by evaporating a silver layer on the dewetting structures (see “Fabrication of SERS substrate” in methods), just like shown in Fig. 5a. The sphere consisting of silver shell and polymer droplet core can be seen as the silver nanosphere. We can easily control the diameter and gap spacing of silver nanospheres by changing the diameter and gap spacing of polymer droplets. We evaporate silver atoms on the PS/PMMA blend films and PS or PMMA film with the same original thickness (h = 8 nm) to fabricate the 3.5 cm × 3.5 cm large area SERS substrates, referred to 8 nm S0/M100@Ag, 8 nm S20/M80@Ag, 8 nm S50/M50@Ag, 8 nm S80/M20@Ag and 8 nm S100/M0@Ag, respectively. The morphology of 8 nm S50/M50@Ag SERS substrate is shown in Fig. 5b. It is worth mentioning that we can fabricate much larger area SERS substrate using the same method.

To study the SERS performance of these SERS substrates, we drop 10 μl R6G aqueous solutions (10^−6 ^M) on the SERS substrates. The Raman signals of R6G on the different SERS substrates are shown in Fig. 5c. The stronger Raman signal indicates the better enhanced property of SERS substrate. From Fig. 5c, we cannot get the clear Raman signal of R6G on the 8 nm S100/M0@Ag substrate, 8 nm S0/M100@Ag substrate and silicon wafer@Ag substrate. However, using the PS/PMMA blend film to fabricate SERS substrate, we can get the clear Raman signal of R6G and the Raman signal of R6G on the 8 nm S50/M50@Ag SERS substrates is strongest. The Raman signals of R6G result from C-C-C ring in-plane bending (614 cm^−1^) and out of plane bending (771 cm^−1^), aromatic C-C stretching (1183, 1368,1509, 1575 and 1654 cm^−1^), which is in agreement with the Raman signature of R6G reported in earlier literatures^54^,^55^. Thus, the 8 nm S50/M50@Ag substrate owns the better SERS property. The wide accepted and dominant mechanism behind the SERS is the electromagnetic enhancement mechanism. In the electromagnetic enhancement mechanism, the larger enhancement factor of SERS is due to the enhanced electromagnetic field, where the optical excitations of localized surface plasmons (LSPs) resonances in metallic nanostructure enhance the Raman signal intensity^56^. Under conditions of surface plasmon excitation, both the incident laser field and the scatter Raman field are amplified. According to previous studies about SERS substrates, the size of nanostructures and interparticle gap spacing strongly influence the SERS property of substrates^10^,^11^,^57^. For the 8 nm S50/M50@Ag, the density of the silver nanosphere (or the polymer droplets) is largest (see Fig. 1), the distance of the silver nanosphere is smallest, which makes the electric field strongest^11^. Thus, the S50/M50@Ag substrate owns the best SERS property as original thickness of polymer films is the same.

In order to study the influence of separation spacing (*D*_*s*_) between droplets on SERS property, we compare the SERS properties of S50/M50@Ag, S100/M0@Ag, and S0/M100@Ag with the same diameter of polymer droplets (*D*_*d*_ is about 80 nm) and different droplet separation spacing (*D*_*s*_), the results shown in Supplementary Fig. S7. We can only get the distinct Raman signal of R6G (10^−6 ^M) on the S50/M50@Ag substrate as *D*_*d*_ is 80 nm. As the *D*_*d*_ is 80 nm, the droplet separation spacing (*D*_*s*_) of S50/M50 film is 100 nm and that of S100/M0 film and S0/M100 film are 200 nm. Thus, the smaller separation spacing *D*_*s*_ contributes to better SERS property.

To study the role of the droplet diameter in the SERS property, the SERS properties of S50/M50@Ag, S100/M0@Ag, and S0/M100@Ag with the same polymer droplet separation spacing *D*_*s*_ (*D*_*s*_ is about 200 nm) and different droplet diameter (*D*_*d*_) are also compared, and the results are shown in Supplementary Fig. S8. It is clear that the SERS signal of R6G (10^−6 ^M) on the S50/M50@Ag substrate is stronger. Because the droplet diameter of S50/M50 is bigger than that of S100/M0 and S0/M100, and we can get the conclusion that the bigger droplet diameter contributes to better SERS property.

**Figure 6:**
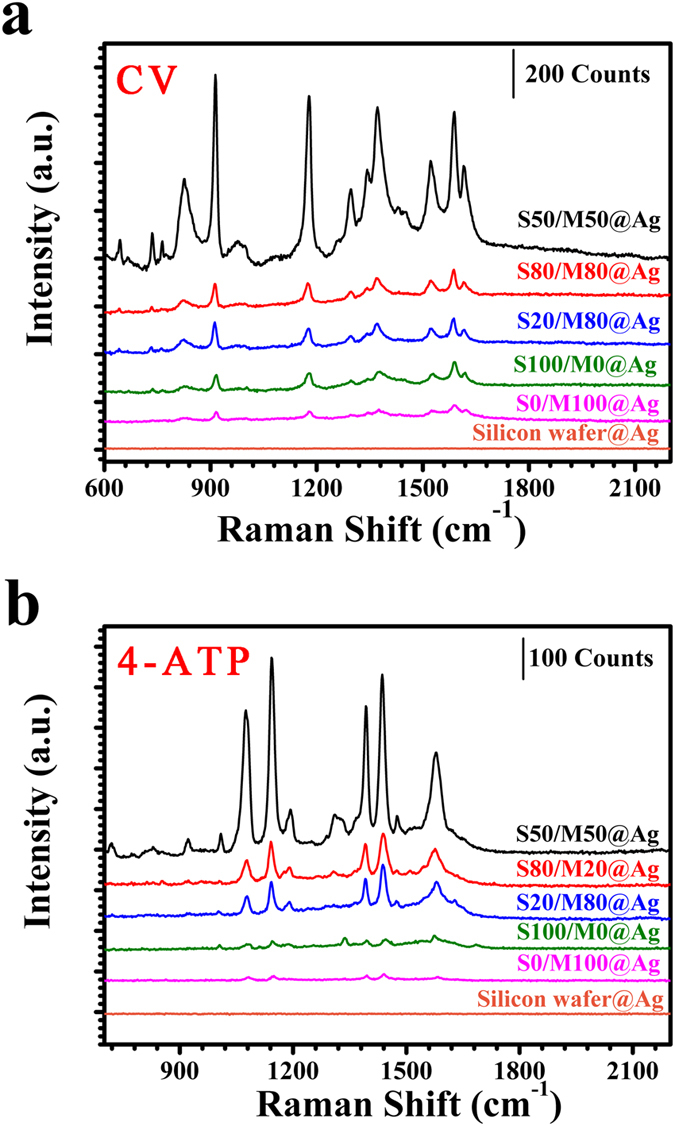
SERS spectra of CV and 4-ATP on the different SERS substrates. The polymer films own the same original thickness (h = 8 nm). (**a**) SERS spectra of CV (10^−6 ^M) on the different SERS substrates; (**b**) SERS spectra of 4-ATP (10^−5 ^M) on the different SERS substrates.

From Figs 1 and 4a, S50/M50 film produces droplets with smaller separation spacing than single PS film or PMMA film with same thickness. Thus, the dewetting of polymer blend films has distinct advantage than the dewetting of single Polymer film in fabricating the SERS substrate. Based on the above result, we can hold that the S50/M50@Ag substrate owns the best SERS property. By calculation, the enhancement factor (*EF*) for R6G of 8 nm S50/M50@Ag substrate is 2.5 × 10^7^ at the peak of 614 cm^−1^ (see the section“SERS enhancement factor (*EF*)” in methods). A 30 × 30 μm area on the 8 nm S50/M50@Ag substrate is mapped by point-by-point scanning with a step size of 5 μm (6 × 6 spots) during laser excitation. Figure 5d is the two-dimensional Raman mapping at the R6G (10^−6 ^M) Raman peak of 614 cm^−1^. There are 36 pixels (6 × 6) and every pixel shows the similar peak intensity. The Raman signals of 36 spots are shown in the Fig. 5e. Figure 5e presents these signal intensities at 614 cm^−1^ of R6G (10^−6 ^M) and the relative standard deviation (*RSD*) of these SERS intensities is 7.2%. Finally, to confirm that this enhancement effect is universal, we have also measured other different molecules, including CV and 4-ATP. Figure 6 also shows that the Raman signals of CV and 4-ATP on 8 nm S50/M50@Ag substrate are strongest. In a word, the enhanced dewetting of ultrathin polymer blend film can be used to successfully fabricate large-area, uniform, low-cost, high efficient and universal SERS substrate.

## Conclusion

We investigate the dewetting of ultrathin PS/PMMA blend films under mixed solution of methyl ethyl ketone (MEK), acetone and water, and fabricate the larger-scale SERS substrate by using the dewetting structure of PS/PMMA blend film. It is proved that the enhanced dewetting method of ultrathin PS/PMMA blend films can precisely control the diameter of droplets, diameter distribution of droplets and the average separation spacing between the droplets. Based on the results of selective etching and the thermodynamics analyses, the PS/PMMA firstly undergo vertical phase separation and form a bilayer structure consisting of the under PMMA-rich layer and the upper PS-rich layer during the spin-coating process. The instability of the thicker layer of the bilayer structure dominates the dewetting structures. As blend ratio of PS/PMMA is 1:1, the diameter of droplet and average separation spacing between the droplets reach minimum, and the diameter distribution of droplets is narrowest. Because, both thicknesses of the PS-rich layer and the PMMA-rich layer are close to half of the blend film thickness as blend ratio is 1:1, and the thickness of layer dominating the dewetting structure is thinnest. The dewetting of 8 nm S50/M50 film under mixed solution can be used to fabricate large area (3.5 cm × 3.5 cm, even larger) SERS substrate via depositing a silver layer on the dewetting structure. The 8 nm S50/M50@Ag SERS substrate displays a high enhancement factor (2.5 × 10^7^) and excellent signal reproducibility (*RSD* < 7.2%). The SERS substrate is valid to several different molecules including R6G, CV, and 4-ATP. In a word, the dewetting of S50/M50 blend film in mixed solution can broaden the application of polymer film dewetting in nanotechnology and is proved to be a simple, low-cost and efficient method to fabricate large area SERS substrate which has the promising potential for application in analytical chemistry and environmental science so on.

## Methods

### Material

PS (M_w_ = 20000 g/mol; M_w_/M_n_ < 1.06), PMMA (M_w_ = 20000 g/mol; M_w_/M_n_ < 1.07), Rhodamine 6 G (R6G), Crystal Violet (CV) and 4-Aminothiophenol (4-ATP) were purchased from Sigma-Aldrich. Analytical reagent (*A.R.*) Toluene, acetone and methyl ethyl ketone (MEK) were purchased from Beijing Chemical Works. These materials were used directly without purified. Silicon wafer (P<100>, 0.3–50 Ωcm) was supplied by Wafer Works Shanghai Corp.

### Preparation of ultrathin polymer blend film

A series of toluene solution of PS/PMMA (100:0, 80:20, 50:50, 20:80, 0:100 wt%) were spin-coated on HF-treated silicon wafers at 3000 rpm for 30 s to prepare ultrathin polymer film. For simplicity, these films were referred to as S100/M0, S80/M20, S50/M50, S20/M80, S0/M100, respectively. The thicknesses of these PS/PMMA blend films were changed by varying the concentration of polymer solution. Before spin-coating, the Si wafers were cleaned by acetone and de-ionized water, boiled in a 2/1 (v/v) solution of 98% H_2_SO_4_/30%H_2_O_2_ for 60 min at 100 °C, immersed in a 1/1(v/v) solution of HF/de-ionized water for 60 min at room temperature, finally cleaned by de-ionized water, and dried with compressed nitrogen. After spin coating, the films were stored in the vacuum oven for 24 h at room temperature to remove the residual solvent in the films.

### Dewetting Methodology

The ultrathin PS/PMMA blend films with different blend ratio were put in a chamber containing the mixed solution of methyl ethyl ketone (MEK), acetone and water with the ratio 7:3:35 at room temperature. To increase the dewetting velocity, the amount of water would be reduced appropriately.

### Fabrication of SERS substrate

We vertically deposited 30 ± 5 nm silver film on the completed dewetting PS/PMMA blend films (S100/M0, S80/M20, S50/M50, S20/M80, S0/M100) and silicon wafer by thermal evaporating through a shadow mask in a vacuum of better than 7 × 10^−4^ Pa and a current value of 100A.

### Characterization

The thicknesses of polymer films were measured by ellipsometry (MM-16, France, HORIBA Jobin Yvon). The film morphology, dewetting morphology and thickness of silver film were measured by XL-30 Field Emission Scanning Electron Microscope (FESEM, Japanese, FEI). The diameter and density of droplets were got by ImageJ software. The morphologies of films were studied using a SPA-300HV AFM (Japan, Seiko Instruments Inc.) driven in tapping mode. A silicon tip (OLTESPA-R3, Germany, Bruker) with a spring constant of 2 N·m^−1^ was used.

### Raman measurement

All of the Raman measurements were performed with commercial confocal Raman microscope system (LABRAM HR 800, Horiba Jobin-Yvon) equipped with Leica microscope (50x objective) at room temperature. Laser (532 nm) was used as the light source for the excitation. 10 μl dye solution (R6G aqueous, CV aqueous, and 4-ATP ethanol solution) with different concentrations were dropped on the SERS substrates and were dried for 2 h in clean beach at room temperature. And then the samples were measured by LABRAM HR 800. D2 filter was used, and the exposure time was 5 s, the accumulation number was 1.

### SERS enhancement factor (*EF*)

The SERS *EF* was a quantitative measurement of the Raman signal amplification of the analyte on different SERS substrates, which was calculated by the formula:


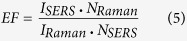


where *I*_*SERS*_ and *I*_*Raman*_ was the SERS intensity and the un-enhanced normal signal intensity, respectively. *N*_*SERS*_was the number of probed molecules in SERS, and *N*_*Raman*_ was the number of probed molecules in normal Raman measurements. Here, a certain volume (*V*_*SERS*_) of R6G aqueous solution (concentration was *C*_*SERS*_) was dropped to an area (*S*_*SERS*_) on the 8 nm S50/M50@Ag SERS substrate. For the normal Raman measurement, a certain volume (*V*_*Raman*_) of R6G aqueous solution (concentration was *C*_*Raman*_) was dropped to an area of *S*_*Raman*_ on a clean glass wafer. Both the R6G aqueous solutions were dried in the air at room temperature. The area of laser spot was same in the SERS measurement and normal Raman measurement. Thus, the foregoing equation (5) became^58^,^59^:


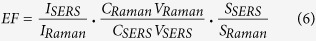


In the SERS measurement, 10 μl of 1 × 10^−6 ^M R6G aqueous solution were dispersed to an circular area of 12.56 mm^2^ for the S50/M50@Ag SERS substrate. In the normal Raman measurement, 10 μl of 1 M R6G aqueous solution were dispersed to an circular area of 28.26 mm^2^ for the glass wafer. For the band at 614 cm^−1^, *I*_*SERS*_/*I*_*Raman*_ was 1896/33 (see Supplementary Fig. S9). Therefore, the *EF* is 2.5 × 10^7^.

## Additional Information

**How to cite this article**: Zhang, H. *et al*. Enhanced Self-Organized Dewetting of Ultrathin Polymer Blend Film for Large-Area Fabrication of SERS Substrate. *Sci. Rep.* **6**, 38337; doi: 10.1038/srep38337 (2016).

**Publisher's note:** Springer Nature remains neutral with regard to jurisdictional claims in published maps and institutional affiliations.

## Supplementary Material

Supplementary Information
