# Supplementary material for: Enhanced Self-Organized Dewetting of Ultrathin Polymer Blend Film for Large-Area Fabrication of SERS Substrate
Source: Sci Rep. 2016 Dec 6;6:38337. doi: 10.1038/srep38337 (PMC5138605; doi:10.1038/srep38337)
Supplement: Supplementary Information [file srep38337-s1.pdf]

Supplementary Information

Enhanced Self-Organized Dewetting of Ultrathin Polymer Blend Film for Large-Area Fabrication of SERS Substrate

Huanhuan Zhang [1,2,3], Lin Xu*[,2], Yabo Xu[1,3], Gang Huang[1,3], Xueyu Zhao[4], Yuqing Lai[1] & Tongfei Shi*[,1]

[1]State Key Laboratory of Polymer Physics and Chemistry, Changchun Institute of Applied Chemistry, Chinese Academy of Sciences, Changchun 130022, P. R. China;

[2] Laboratory of Surface Physics and Chemistry, Guizhou Education University, Guiyang 550018, P. R. China;

[3] University of Chinese Academy of Sciences, Beijing 100049, P. R. China.

[4] School of Chemistry and Life Sciences, Guizhou Education University, Guiyang 550018, P. R. China.

*Correspondence to: lxu@gznc.edu.cn (L.X) and tfshi@ciac.ac.cn (T.F.S)

This PDF file includes: Figure **S1** to **S9**

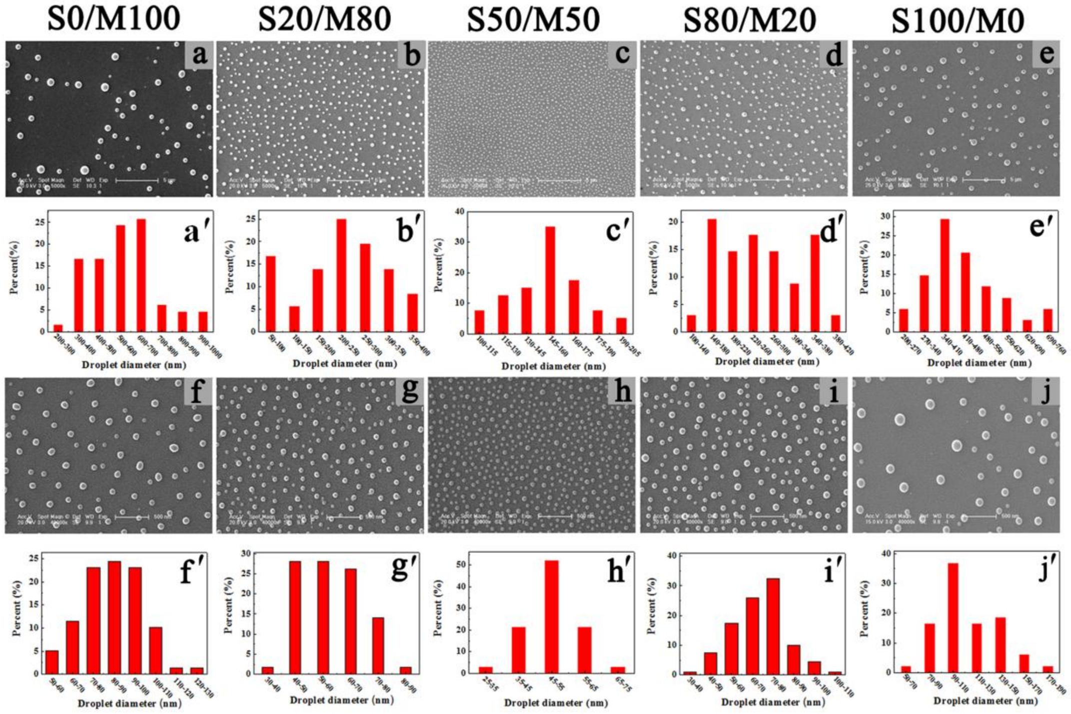

Figure S1. FESEM images of polymer blend films with different thickness after dewetting in the mixed solution. (a)- (e) the thickness of films is about 17nm. (f)- (j) the thickness of films is about 5 nm. (a′) - (e′) and (f′) - (j′) are the diameter distribution of the (a) - (e) and (f) - (j) respectively. The 5nm films were put in the mixed solution (MEK: Acetone: Water 7:3:35) for 10 minutes to induce dewetting. To increase the dewetting velocity, the 17nm polymer blend films were put in the mixed solution containing less water (MEK: Acetone: Water 7:3:25) for 24 h to induce dewetting. The scale bar in (a)-(e) is 5μm and that in (f)-(j) is 500 nm.

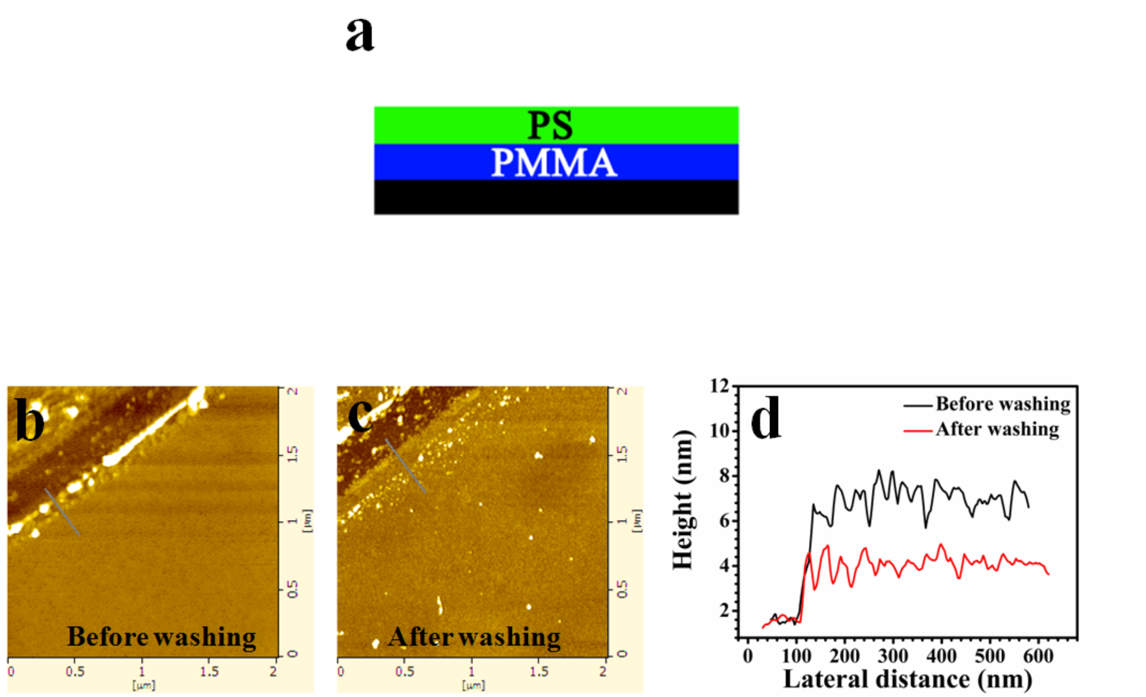

Figure S2. (a)The schematic diagram of bilayer film consisting of under PMMA film and upper PS film. Firstly, the PMMA layer was prepared by spin-coating the toluene solution of PMMA on HF-treated silicon wafer. And then the PMMA film was put in vacuum oven for 24h at room temperature to remove residual solvent. Finally, the PS layer was prepared by spin-coating the cyclohexane solution of PS on the PMMA layer; (b) AFM images of the bilayer film before washing by cyclohexane; (c) AFM images of the bilayer film after washing by cyclohexane; (d) the sectional views along the line (the gray line) in (b) and (c).

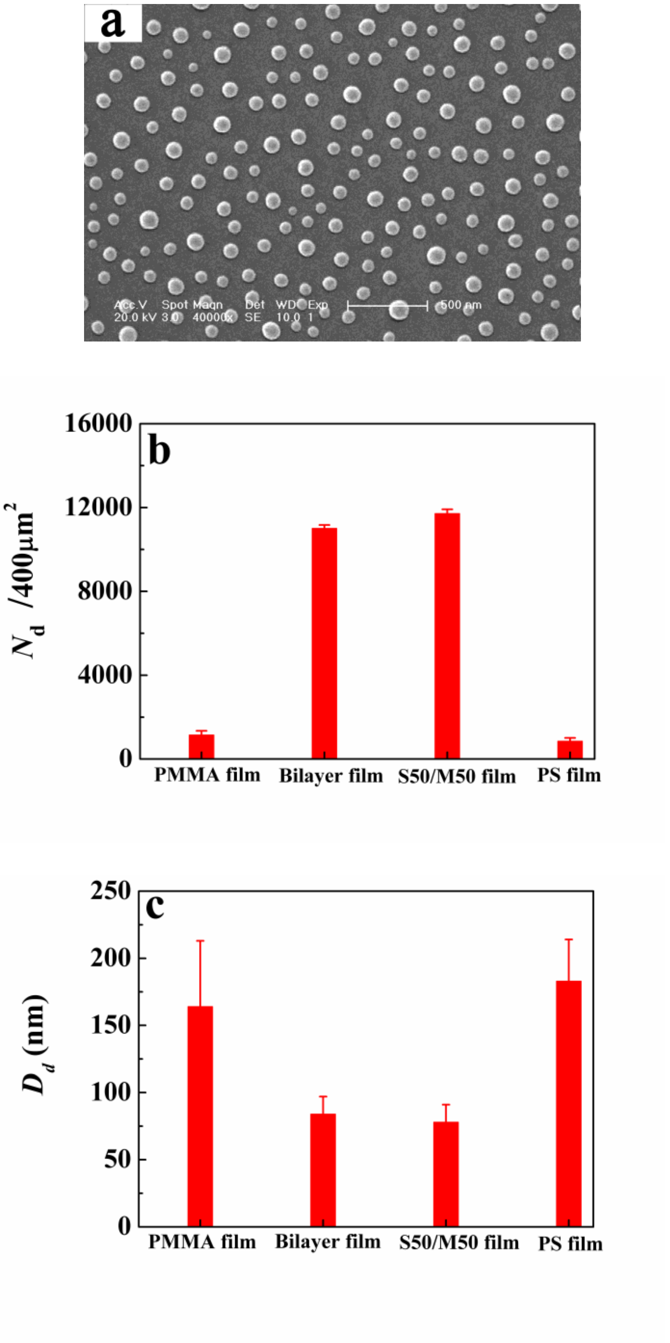

Figure S3. The dewetting results of bilayer film. (a) the final morphology of dewetting droplet for bilayer film;(b) and (c) the number (N_d) and diameter (D_d) of droplets for bilayer film, polymer blend film (or S50/M50 film) and single polymer film. The film thicknesses of bilayer film, polymer blend film and single polymer film are same, 8nm.

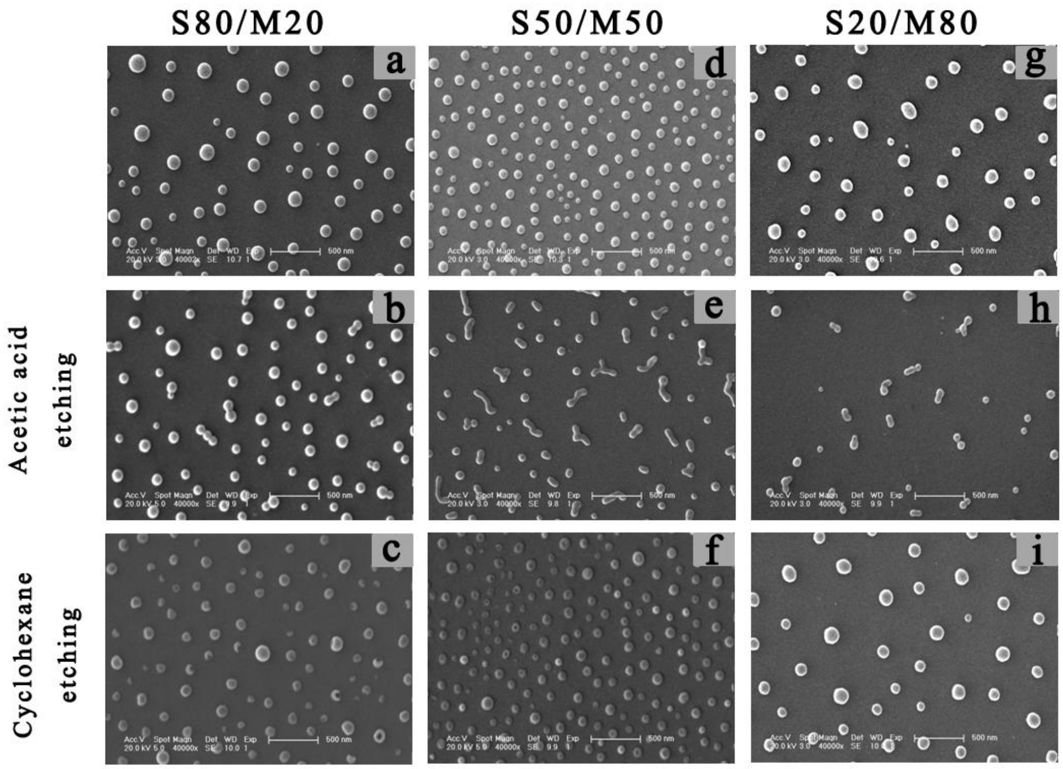

Figure S4. FESEM images of dewetting droplets without or with etching. The images in the first row are the morphologies of raw dewetting droplets. The images in the second row and third row are the morphologies of droplets etched by acetic acid and cyclohexane for 10 min, respectively. (a)-(c) 8 nm S80/M20 film; (d)-(f) 8 nm S50/M50 film; (g)-(i) 8 nm S20/M80 film. Scale bar is 500 nm.

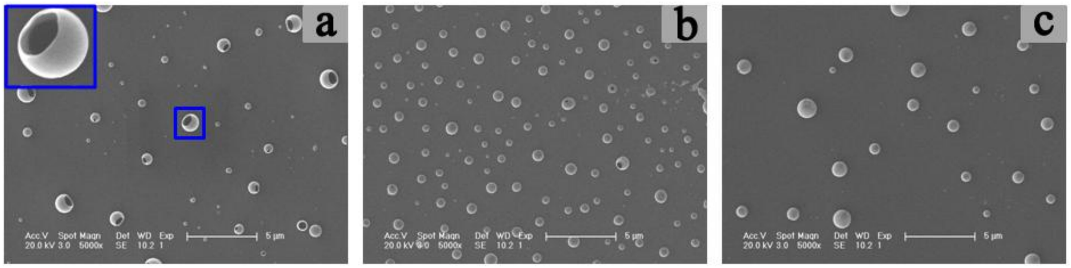

Figure S5. FESEM images of dewetting droplets etched by cyclohexane for 10min. The original thickness of films is 27 nm. The polymer blend films were put in the mixed solution of methyl ethyl ketone (MEK), acetone and water in the ratio 7:3:15 to induce dewetting at room temperature. (a)-(c) the films are S80/M20, S50/M50, S20/M80, respectively. The inset picture in (a) is the magnification of one etched droplet. For S80/M20 films, the morphology of etched droplet is nano-bowl. In the case of S50/M50, the holes in the etched droplets are smaller and fewer. For the S20/M80 film, the etched droplets are still the compete droplets. As PMMA fraction is below 50%, the PMMA cannot totally encase the PS core. On the contrary, the PMMA can totally encase the PS core.

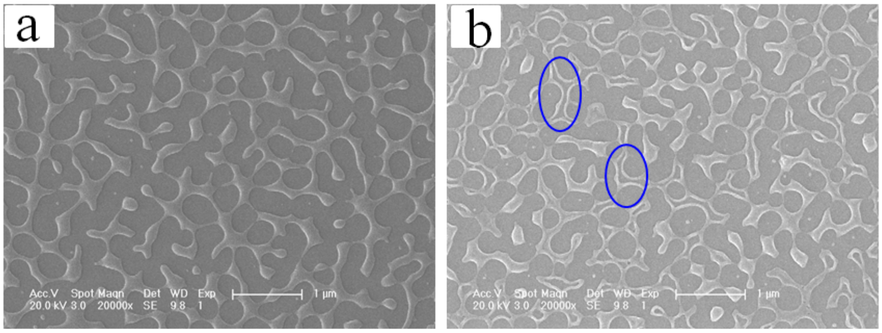

Figure S6. FESEM images of dewetting droplets without (a) or with (b) etching via cyclohexane (27 nm S50/M50 film). The blue circles in (b) are guides to eyes.

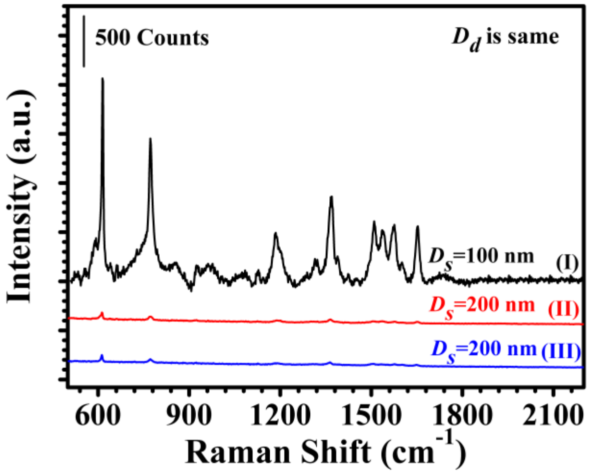

Figure S7. SERS spectra of R6G (10^{-6}M) on the different SERS substrates with the same droplet diameter (D_d = 80 nm) and different droplet separation spacing (D_s). (I)- (III) are S50/M50@Ag, S100/M0@Ag and S0/ M100@Ag, respectively. The original thickness of S50/M50 films is 8nm and that of S100/M0 film and S0/M100 film are 5nm.

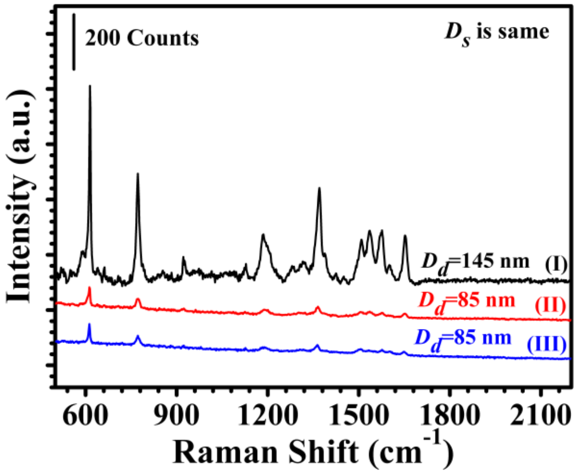

Figure S8. SERS spectra of R6G (10^{-6} M) on the different SERS substrates of the same droplet separation spacing (D_s = 200 nm) and different droplet diameter (D_d). (I)- (III) are S50/M50@Ag, S100/M0@Ag and S0/M100@Ag, respectively. The original thickness of S50/M50 films is 21nm and that of S100/M0 film and S0/M100 film are 5nm.

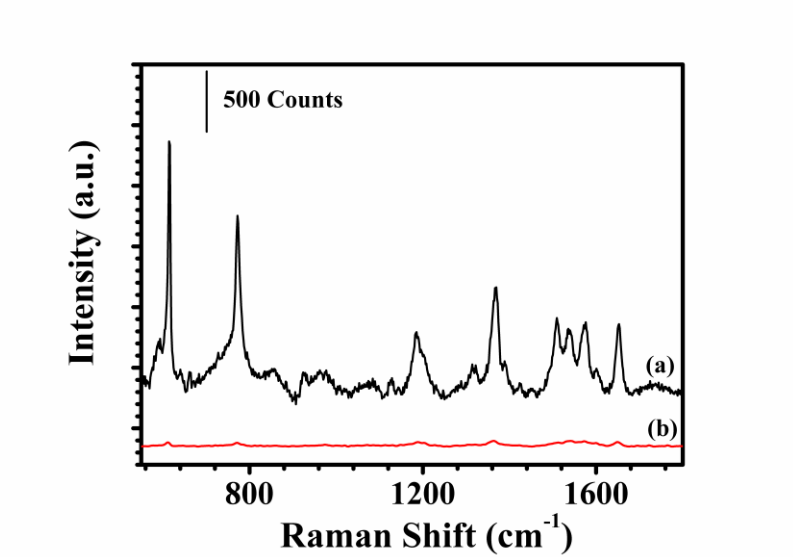

Figure S9. The Raman spectrum used to calculate the EF. (a) SERS spectrum of 10 μl R6G aqueous solution(1×10^{-6}M) was dispersed to an circular area of 12.56 mm^2 for the 8 nm S50/M50@Ag SERS substrate. (b) Raman sectrum of 10 μl R6G aqueous solution (1M) was dispersed to an area of 28.26 mm^2 for the glass wafer.
